# Clinical Lectures in the Medical Wards of the Illinois General Hospital

**Published:** 1851-09

**Authors:** N. S. Davis

**Affiliations:** Prof. of Pathology, Practice, and Clinical Medicine, in Rush Medical College


THE NORTH-WESTERN
MEDICAL AND SURGICAL JOURNAL.

VOL. IV.] SEPTEMBER, 1851. [No. 3.

### Part 1.—Original Communications.

ARTICLE I.

*Clinical Lectures in the Medical Wards of the Illinois General Hospital.* By N. S. DAVIS, M. D., Prof. of Pathology, Practice, and Clinical Medicine, in Rush Medical College.

*Mauch* 2, 1851, 9 *o'clock, A. M.*—Gentlemen: In calling your attention to the case before us, it is proper to remark that the patient, an Irish woman aged 35 years, the mother of several children, the youngest of whom is but a few months old, was seen by me two days since, when I found her in a small house destitute of every thing required for the comfort or welfare of the sick, and hence I procured her removal to the Hospital, where she was admitted last evening. It appears, from what I have been able to learn in regard to her history, that she was attacked about ten days since with the symptoms of ordinary remittent fever, viz: chills, followed by pain in the head and back, with a hot and dry skin, distinctly remitting every morning and returning with increasing intensity each afternoon. After the first three or four days, the exacerbations of fever were accompanied by delirium and great restlessness, and the morning remissions were less distinct. When I saw her two days since, her face was deeply flushed, head hot, skin hot and dry, tongue covered with a brownish yellow fur, dry, with some redness of the tip and edges, pulse 100 per minute and moderately full, much thirst, eyes injected and watery, bowels torpid, abdomen full, and respiration hurried. She was directed to take a powder composed of Calomel 3 grs., Ipecac, 1 gr., Sup. Carb. Soda 3 grs. every three hours until four doses had been taken, and then follow them with a table-spoonful of Castor Oil; the head to be kept cool by cloths dipped in cold water, and sinapisms applied to the feet. The castor oil was followed by two or three copious evacuations from the bowels, and an almost entire intermission in the febrile symptoms, during which she was ordered Sulph. Quinine 15 grs., Pulv. Opii. 1 gr., to be divided into three doses, and given one every three hours until all were taken. Owing to the want of attendance, however, this medicine was not given, and the patient was transferred to the Hospital.

Such is a brief history of the case up to the present time. You will now observe that the patient presents a very dull and stupid expression of countenance, the conjunctiva of the eye is injected, the position is dorsal, the prolabia and cheeks of a leaden or purplish hue, skin dry and warm, tongue covered with a brownish coat in the middle and red at the edges and tip, much thirst, abdomen full and moderately tympanitic, has had during the last twelve hours two or three dark, fœtid and liquid evacuations from the bowels, pulse 110 per minute and easily compressed, little or no milk in her breasts, and manifests entire indifference in regard to her children. She complains of a disagreeable ringing or noise in her head, and slight deafness. The respiration is quicker and shorter than natural, and as I place my ear over the infra-clavicular regions, a dry whizzing or sibilant rattle is heard over a considerable extent on both sides. But the resonance on percussion is good.

Such, gentlemen, are the present symptoms. What do they indicate? How shall we interpret them, and what is the true pathological condition of the patient? In technical language, what is the diagnosis? The past history shows it to have been in the beginning a distinct remittent, arising under circumstances of exposure, destitution and mental anxiety. This continuing unchecked by appropriate treatment, gradually exhausted the vital energies on the one hand, and developed a phlogistic or inflammatory condition of the mucous membranes on the other. Hence the frequent and compressible pulse, the mental indifference, and stupid expression of countenance, coupled with the full and tympanitic condition of the abdomen, the dark watery evacuations, and the dry sibilant zonchus, which now constitute the prominent features of the case, and fairly bring it under the head of Typhoid fever, with special local determinations. The word Typhoid, as you are well aware, is used by different writers differently; some regarding it simply as synonymous with Typhus, while others, among whom are many eminent French writers as well as some in our own country, apply it to what they regard as a distinct variety of fever characterized by specific pathological lesions. I think, however, that the words Typhus and Typhoid should be used as strictly comparative terms, indicating different degrees of the same general condition of the organic or vital forces, and without reference to any special local lesions. For instance, if disease attacks an individual poorly fed, and exposed to the foul air of damp, ill-ventilated apartments, or the crowded hold of a ship, his symptoms will at once present that peculiar aspect which all agree in representing as true Typhus. And if it proceeds to a fatal termination, a post mortem examination may reveal local lesions either in the head, the chest, or the abdomen. True, there is a greater tendency to develope local disease in the latter cavity, and especially in the mucous membrane of the intestines than elsewhere; but this by no means occurs so uniformly as to be entitled to the appellation of a specific and essential part of the disease. Again an individual exposed to less depressing and poisonous influences, may be attacked with fever either remittent or continued, and during the first week the symptoms will present a more or less active or stenic character. But the disease continuing unchecked, the excretory organs, as the skin, kidneys, liver, &c., perform their functions but imperfectly, leaving more or less excretory matter of a deleterious and depressing nature in the system, which added to the ordinary exhausting effects of all morbid action, sooner or later induces that impaired condition of the organic or vital forces which I have already described as characteristic of true Typhus. In ordinary professional parlance, what was regarded in the beginning as a simple remittent or continued fever, has now put on a Typhoid form. And this Typhoid state may, like true Typhus, be accompanied by local lesions in any of the important viscera of the system, though experience has shown that the folicles and glands of the intestinal mucous membrane are by far the most frequent seat of such lesions. You will thus see, gentlemen, that I use the words Typhus and Typhoid, to indicate a certain impaired condition of the vital forces in a febrile state, without reference to any particular local affection, or the intervention of any specific cause. Taking this view, you will readily see that we may have in practice every shade of Typhoid condition, from the complete Typhus which occurs among emigrants crowded on shipboard, to the slightest Typhoid tendency induced as a result of a more active but uncontrolled morbid condition.

In the case before us, we have, strictly speaking, a secondary Typhus supervening on a more active form of fever, and complicated with inflammatory action in the mucous tissue both of the small intestines and bronchial tubes.

*Prognosis.*—The final result of this case is doubtful. The frequency of the pulse, and the evidences of local disease, both in the pulmonary and intestinal mucous membranes, with the well known tendency of the latter to terminate in extensive superficial ulceration, give us fears of a fatal result. Still many cases recover with symptoms more unfavorable than in the present instance, and hence the patient ought to be encouraged to hope for the best.

*Treatment.*—You will see at a glance that we have two prominent indications to fulfill in the further treatment of this case, viz: First, to sustain the circulation and nervous energy of the system, or, at least, prevent them from becoming further impaired; and second, to remove the local affection of the mucous surfaces. What we have always to fear in such cases as this, is, that the intestinal mucous surface will become ulcerated, causing the abdomen to become more tympanitic, and the discharges more frequent and watery or dark and grumous, with steadily increasing depression of the vital forces; or the pulmonary mucous surface will take on a copious suppurative action, inducing copious expectoration, night sweats and rapid emaciation. Hence the accomplishment of the second indication named is the all-important object at the present moment. But how shall this be done? Not by depletion either general or local—not by mercurials or antimony; for there is already too much depression to admit any of these, except perhaps an alterative dose of Calomel given at remote intervals and coupled with an anodyne. Neither will the patient be benefited by that class of stimulants called diffusive, and which tend to excite the circulation. In my own practice I have found no class of remedies to act as favorably on that peculiar grade of mucous inflammation, (if such we may call it,) which exists in this and similar cases, as the Balsamic and Terebinthinate articles conjoined with small doses of opiates, aided by counter irritation externally. Without attempting to explain the exact *modus operandi* of this class of remedies, we may suppose that they exert a peculiar influence over the capillary circulation, while they promote the secretions from the skin and kidneys without depressing the general tone or energy of the organic actions. They are best given in the form of emulsion. At present we will direct the following formula: ℞ Balsam Copaiba ʒij, Tinct. Opii. ʒj, White Sugar and Gum Arabac each ʒiij, rubbed thoroughly together and added to Water ℥ij. Of this one tea-spoonful must be given every four hours. We will also place a large blister over the epegastric region, and allow bread water, with some plain animal broth for drink and nourishment.

*March* 3, 9 *o'clock, A. M.**—All the symptoms remain as yesterday, except the tongue is more moist, the dry ronchus of yesterday is changed to one more moist, with an occasional cough and expectoration of muco-purulent matter, and no alvine evacuations since last visit. Blister has drawn well. The pulse remains over 110 per minute, and the abdomen more tympanitic.

Ordered a cathartic composed of Castor Oil two parts, Oil of Turpentine one part, half an oz., and after its operation to continue the emulsion every six hours.

*March* 4, 9 *o'clock, A. M.*—The cathartic procured three copious, dark-colored, but less watery evacuations from the bowels; the abdomen is much less tympanitic, the tongue moist and less red at the tip and edges, the skin moist, and expression of countenance somewhat improved. Still, the pulse remains as frequent as before, and the cough and expectoration have greatly increased.

Continue the emulsion as before, with another blister over the upper and anterior part of the chest.

*March* 5, 9 *o'clock, A. M.*—The symptoms of intestinal

---

*Having embodied a pretty full view of the case in the report of yesterday, we shall omit the Professor's remarks from day to day, and report only so much as will enable the reader to follow the treatment to its termination.

irritation have almost entirely subsided. There has been one evacuation from the bowels, and that of a more consistent and natural appearance. But the patient had a distinct febrile paroxysm last evening, which ended towards morning in a profuse sweat, the skin still remaining moist and the patient complaining of much weakness. The expectoration still remains copious, of a purulent character, and the pulse 105 per minute. Ordered her Quinine 5 grs., with Pulv. Opium ¼ gr., to be given now, and the same repeated to-morrow morning at 6 o'clock. Also a mixture of Hive Syrup ℥jj, Tinct. of Blood-root ℥ss., and Tinct. Opii ʒj; mix and give one tea-spoonful every four hours.

This treatment was continued with a subsequent additional blister over the chest, and an occasional laxative of Syrup of Rheubarb through the next five days, and the patient steadily though slowly improved. The hectic paroxysms subsided, the expectoration lessened, the pulse became slower, and the tongue clean and natural. The patient, however, remained much emaciated and feeble. She was then put on the use of Cod Liver Oil, a table-spoonful three times a day, with a plain, nutritious diet, and she rapidly recovered her usual health, being discharged on the 1st of April.

